# A Systematic Review of Racial and Ethnic Disparities in Maternal Health Outcomes among Asians/Pacific Islanders

**DOI:** 10.31372/20200503.1101

**Published:** 2020

**Authors:** Janice Hata, Adam Burke

**Affiliations:** Hawai‘i Pacific University, Hawai‘i, United States

**Keywords:** Asians, Pacific Islanders, maternal health, health disparities, obstetrics

## Abstract

Efforts to improve women’s health and to reduce maternal mortality worldwide have led to a notable reduction in the global maternal mortality ratio (MMR) over the past two decades. However, it is clear that maternal health outcomes are not equitable, especially when analyzing the scope of maternal health disparities across “developed” and “underdeveloped” nations. This study evaluates recent MMR scholarship with a particular focus on the racial and ethnic divisions that impact on maternal health outcomes. The study contributes to MMR research by analyzing the racial and ethnic disparities that exist in the US, especially among Asian and Pacific Islander (API) subgroups. The study applies exclusionary criteria to 710 articles and subsequently identified various maternal health issues that disproportionately affect API women living in the US. In applying PRISMA review guidelines, the study produced 22 peer-reviewed articles that met inclusionary and exclusionary criteria for this review. The data analysis identified several maternal health foci: obstetric outcomes, environmental exposure, obstetric care and quality measures, and pregnancy-related measures. Only eight of the 22 reviewed studies disaggregated API populations by focusing on specific subgroups of APIs, which signals a need to reconceptualize marginalized API communities’ inclusion in health care systems, to promote their equitable access to care, and to dissolve health disparities among racial and ethnic divides. Several short- and long-term initiatives are recommended to develop and implement targeted health interventions for API groups, and thus provide the groundwork for future empirically driven research among specific API subgroups in the US.

## Background

Maternal health encompasses the health of women during pregnancy, childbirth, and the postpartum period. Proper maternal care at each of these phases is critical to promoting the health and well-being of women and infants, which is one of the United Nations’ 2015-implemented Sustainable Development Goals (SDGs) (United Nations, 2020b). Pregnancy offers a meaningful opportunity to pinpoint women’s existing health risks and to prevent future health challenges for women, their children, and society at large. Maternal mortality is thus a sentinel event and is used internationally as an indicator of a population’s general health, women’s social status, and the viability of medical systems (Sajedinejad, Majdzadeh, Vedadhir, Tabatabaei, & Mohammad, 2015).

Maternal mortality is a global problem and is measured by the maternal mortality ratio (MMR), which represents the number of maternal deaths per 100,000 live births over a period of time. The MMR captures the obstetric risk, or risk of death associated with each pregnancy or live birth [World Health Organization (WHO), 2006]. UNICEF (2019) informs that the global MMR decreased from 342 in 2000 to 211 in 2017. Despite a 38% reduction in the MMR over that 17-year span (i.e., 2.9% annually), this rate is still less than half the 6.4% annual rate needed to achieve the UN’s aim (e.g., SDG 3, Target 3.1) to reduce the global MMR to less than 70 maternal deaths per 100,000 live births by 2030 (Ritchie, Roser, Mispy, & Ortiz-Ospina, 2018; UNFPA, World Health Organization, UNICEF, World Bank Group, & the United Nations Population, 2019; United Nations, 2020a).

There is significant disparity in MMR data between “developed” and “underdeveloped” countries, which complicates initiatives to reduce the global MMR. For instance, the WHO (2019) found that the regions of sub-Saharan Africa and Southern Asia collectively accounted for about 86% (254,000) of the estimated maternal deaths worldwide in 2017. In the same year, pregnancy and childbirth complications contributed to over 800 daily deaths and MMRs of 533 and 163, respectively (WHO et al., 2019). Conversely, health care systems in many “developed” countries have achieved relatively low maternal death rates, typically ranging from 3 to 12 deaths per 100,000 live births (WHO et al., 2019). The US is the only exception, where the MMR has more than doubled from 1987 to 2018 (7.2 and 17.4 deaths per 100,000 live births, respectively) [Centers for Disease Control and Prevention (CDC), 2020]. According to the CDC (2019), about 60% of pregnancy-related deaths in the US are preventable, exposing a chain of underlying factors impacting on MMR such as access to care and missed or delayed diagnoses.

The two current national data sources for measuring maternal mortality are the CDC’s National Vital Statistics System (NVSS) and the CDC’s Pregnancy Mortality Surveillance System (PMSS). As the official source of maternal mortality data, the NVSS reviews death certificates and ascribes International Classification of Diseases (ICD) codes to identify maternal deaths that occur during pregnancy or within 42 days of the termination of pregnancy, from any cause related to or aggravated by pregnancy or its management, but not from accidental or incidental causes (National Center for Health Statistics, 2007). Alternatively, the PMSS was designed to verify and better understand the causes of pregnancy-related deaths that occurred during or within one year of pregnancy. The PMSS is determined through the clinical analysis of cases and reviews of vital statistics data. Contrary to the NVSS-calculated MMR, the PMSS generates a pregnancy-related mortality ratio (PRMR) which captures the number of pregnancy-related deaths per 100,000 live births, extending the period for detecting maternal deaths from 42 days to one year after childbirth (National Center for Health Statistics, 2007). Despite the PRMR offering a more comprehensive understanding of maternal mortality, this paper utilizes the MMR since it is the metric most commonly identified in the reviewed literature.

Over the past two decades, the MMR in the US has increased despite advances in maternal mortality data. A reason for the increase in maternal deaths is that pregnant women in the US today display chronic health conditions including but not limited to hypertension, diabetes, and chronic heart disease. Studies have shown that women with these conditions experience escalated risk of developing pregnancy complications (Admon et al., 2017; Correa, Bardenheier, Elixhauser, Geiss, & Gregg, 2015; Deputy, Kim, Conrey, & Bullard, 2018). Between 2011 and 2016, the CDC reported that cardiovascular conditions (e.g., cardiomyopathy, cerebrovascular accidents) accounted for more than one-third of pregnancy-related deaths. In addition to chronic disease and pregnancy complications, there are a host of additional risk factors that impact on pregnancy complications, such as advanced maternal age, poor access to obstetric services, and low socioeconomic status (SES) (Howell, 2018).

Maternal outcomes can be conceptualized on a spectrum of severity, beginning with normal and healthy gestation, and extending to morbidity, severe morbidity, and ultimately death. Severe maternal morbidity (SMM) is roughly 100 times more common than maternal mortality and has heightened over recent decades, affecting nearly 60,000 women in the US annually (Howell, 2018). Maternal morbidity is also referred to as “near-misses” and includes unforeseen events during labor and delivery (e.g., uncontrolled bleeding, infection) that result in significant short- or long-term consequences to women’s health (Howell, 2018). During a severe obstetric morbidity, women endure life-threatening pregnancy, delivery, and postnatal complications (e.g., massive hemorrhage, cardiac arrest, organ system failure, stroke) that usually require prolonged hospitalization, major surgery, or other invasive medical interventions (Creanga, Bateman, Kuklina, & Callaghan, 2014).

Considerable racial and ethnic disparities in American maternal mortality trends have existed for more than a century and have increased over time. For example, the CDC’s (2019) MMR data from 2007 to 2016 revealed that black and American Indian/Alaska Native (AI/AN) women experienced significantly higher MMRs (40.8 and 29.7, respectively) than all other racial and ethnic groups (Petersen, 2019). Over the same span, black and AI/AN women (across age groups and all socioeconomic levels) had a two- to three-fold risk of dying from pregnancy-related causes than white women (Petersen, 2019). Similar to maternal mortality rates, racial and ethnic minority women also face increased rates of severe morbidity. For example, Creanga et al.’s (2014) multistate analysis of racial and ethnic disparities in SMM during delivery hospitalizations indicated that SMM rates were higher for black (2.1), Hispanic (1.3), Asian and Pacific Islander (API) (1.2), and AI/AN (1.7) women than white women (1.0), even after controlling for confounding variables (e.g., insurance, income, preexisting conditions). Considering that the US Asian population grew the fastest of any major racial or ethnic group from 2000 to 2015 (e.g., Lopez, Ruiz, & Patten, 2017), it is critical to further explore how and to what extent Asian women are included in prevailing maternal health research.

## Objectives

Amid global efforts to achieve the UN’s ambitious goal of achieving a sub-70 MMR by 2030, and the general trend in MMR research to use nationalism (i.e., political boundaries) to delineate data sets, there is a pressing need to nuance maternal outcomes research in ways that address racial and ethnic disparities in maternal outcomes. The purpose of this paper is thus to explore the racial and ethnic disparities in maternal outcomes research, particularly among API subgroups in the US, which are rapidly growing populations and understudied in health disparities research. A review of maternal outcomes studies on API women is herein offered to recommend effective and targeted health interventions for API communities.

Women of racial and ethnic minorities are disproportionately burdened by higher MMRs and are more likely to experience comorbid conditions and pregnancy complications compared to white women. Studies such as Guendelman, Thornton, Gould, and Hosang (2006) indicate that postpartum hemorrhage (PPH) rates, third- and fourth-degree lacerations, and major puerperal infections are higher among Asian women than white women. Data also demonstrate that women of racial and ethnic minorities develop such conditions at earlier ages, are less likely to have their conditions sufficiently managed, and are more likely to endure complications and die from these conditions than white women (e.g., Beckie, 2017). Notably, studies have shown that the increased risk of maternal mortality across racial and ethnic minority women is partly independent of sociodemographic status (Berg, Chang, Callaghan, & Whitehead, 2003). The racial disparity in pregnancy-related death is not entirely explained in most studies even after adjusting for sociodemographic and reproductive factors.

Consistent disparities across racial and ethnic minorities exist at all junctures of maternal health, including the quality of the delivery hospital, antenatal care, preconception care, and postpartum care. Early and comprehensive antenatal care involves screening and management of risk factors while supporting behaviors conducive to maternal health. Howell (2018) suggested that maternal death and SMM are linked to few or no prenatal visits, and that the initiation and type of prenatal care vary significantly by race and ethnicity. In 2012, white (79%) and Asian (69%) women showed the highest prenatal care initiation rates while black (64%), AI/AN (59%), and Native Hawaiian/other Pacific Islander (NHOPI) (55%) women had the lowest rates (Antony & Dildy, 2013). Perinatal research studies (e.g., Gadson, Akpovi, & Mehta, 2017) primarily focus on racial and ethnic minorities’ experiences of racism, discrimination, and stress and role these experiences have in delayed or inadequate prenatal care. However, additional studies are needed to understand these experiences and their association with maternal outcomes.

National approaches to deal with and reduce maternal mortality and severe morbidity generally strain health care systems and struggle to account for and serve marginalized populations. Socioeconomic inequalities and state-level policies often have a magnified impact on women’s health when compared to men’s health. For instance, access to essential services and resources (e.g., antenatal care, children’s health care) are pivotal in women’s health (Vilda, Wallace, Dyer, Harville, & Theall, 2019). Vilda et al. (2019) emphasize that women are generally more vulnerable to the detrimental consequences of income inequality and inequitable distribution of public resources. The CDC’s NVSS 2018 report on race differences in maternal mortality determined that black women in America have the highest MMR (37.1), followed by white women (14.7), and Hispanic women (11.8). Data for API women were not reported—and thus highlights a reality that health disparities research among API populations in the US are limited. Moreover, APIs are often aggregated into a single homogenous group, which consequently obscures distinctive disparities across subgroups.

The study of maternal health disparities among API populations is thus an area needing additional scrutiny. Accordingly, this paper contributes to API scholarship by systematically examining the recent maternal health disparities literature on API populations in the US, to make sense of recent federal efforts to enhance maternal health disparities research. The findings from this review have the potential to positively impact local and global engagement with maternal mortality studies and to further promote the health and well-being of all racial and ethnic subgroups within the broad API label.

Methods: Systematic Review, Data Sources, Inclusion/Exclusion Criteria, and Number of Eligible Sources for Systematic Review

**Figure 1.**
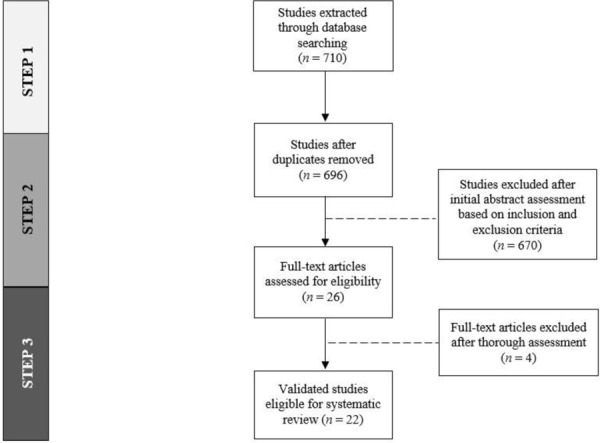
PRISMA flow diagram.

This paper utilizes content analysis to systematically review themes, patterns, and biases in 710 maternal health studies that were extracted from three online databases. Figure 1 illustrates a PRISMA flow diagram of the process for identifying studies for this review. The analysis examines trends in maternal outcomes (e.g., PPH, pregnancy-related complications, deficient obstetric care) and highlights various health issues that disproportionately affect API women living in the US.

In Step 1, a search of existing literature was conducted across three online databases: PubMed, EBSCO, and ScienceDirect. The search identified recent and relevant articles to be included in the analysis. The primary terms “Asian,” “Pacific Islander,” “maternal health,” “maternal mortality,” “maternal morbidity,” and “racial and ethnic disparities” were searched in each database. The database searches also included other relevant terms “perinatal,” “pregnancy,” “health care,” and “quality” to narrow the results. The three database search yielded 710 total articles. After removing duplicate articles, 696 unique studies remained for potential inclusion in the analysis.

In Step 2, the 696 studies were screened, and abstracts were evaluated according to the inclusion and exclusion criteria developed specifically for this review. Articles were *included* in the review if they (i) had a substantial focus on Asians and/or Pacific Islanders residing in the US; (ii) focused on racial and ethnic disparities in maternal health outcomes, including differences in maternal risk factors and obstetric quality and safety; (iii) used data-driven methods to identify or evaluate maternal health disparities among API groups; and (iv) were published between 2015 and 2020. Articles were *excluded* in this review if they (i) primarily studied populations other than Asians and/or Pacific Islanders and the research was not based in the US, (ii) focused chiefly on disparities in neonatal or infant health outcomes, (iii) used nonempirical methods (i.e., theoretical in nature), (iv) were published prior to 2015, and (v) were not peer-reviewed. This initial filtering of article attributes reduced the number of acceptable full-text articles to 26.

In Step 3, the 26 full-text articles were assessed for eligibility and validated through an additional round of screening across the research methodologies and results to determine if the studies merited inclusion in this analysis. This step revealed that several articles lacked empirically based methods or did not focus on API groups in the US. These studies were removed from inclusion in the review, reducing the number of eligible full-text articles to 22. See Table A1 in the Appendix for an overview (e.g., authors, title, issue, *N*, study design, major findings) of the 22 eligible full-text articles that inform the results in the next section.

**Table A1:** An Overview of the 22 Eligible Full-Text Articles Produced During the Literature Review. Data from these Studies were used to Inform the Key Themes Identified in the Results Section

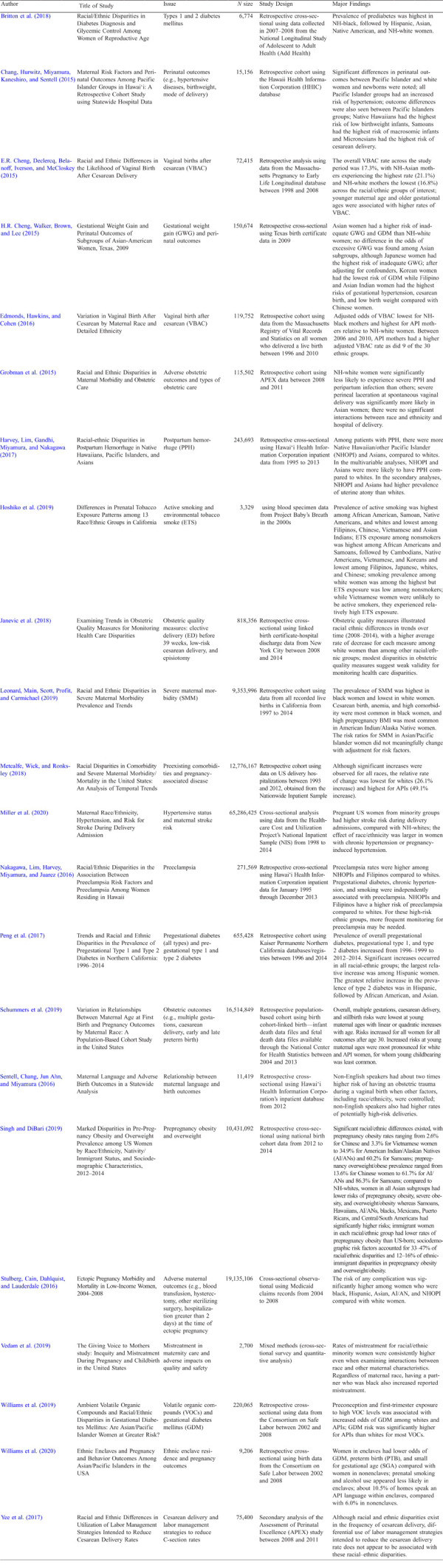

## Results

The 22 peer-reviewed studies that met inclusion and exclusion criteria for this review are summarized in Table A1. The reviewed studies were categorized by maternal health focus: 41% of the articles focused on obstetric outcomes, 13% focused on environmental exposures, 23% focused on obstetric care and quality measures, and the remaining 23% focused on risk factors for pregnancy-related complications. Only 36% of the 22 reviewed studies disaggregated API populations by focusing on specific subgroups (e.g., Chinese, Japanese, Filipino, Korean, Samoan, Micronesian, Native Hawaiian). Of the reviewed articles, 82% occurred across the continental US and 18% occurred exclusively in the State of Hawai‘i. All but one study (Vedam et al., 2019) relied on preexisting data sources (e.g., Consortium on Safe Labor, Medicaid records, linked birth certificate-hospital discharge data) to retrospectively conduct the research. All of the reviewed studies were analytical (observational) in nature and included large sample sizes.

As regards obstetric outcomes (e.g., blood transfusion, preeclampsia, hemorrhage), these results further support the notion that API groups are significantly impacted by adverse maternal outcomes. Nine studies in the review primarily addressed specific maternal outcomes and generally found that APIs—and other racial and ethnic minorities—faced an increased risk of developing adverse outcomes. Notably, Grobman et al. (2015) found significant racial and ethnic differences in the frequency of various maternal morbidities. White women were least likely to experience severe PPH or peripartum infection and Asian women were most likely to experience a severe perineal laceration at spontaneous vaginal delivery and had significantly greater odds of experiencing a severe PPH or peripartum infection than white women. These racial and ethnic disparities remained true even after controlling for other patient characteristics [e.g., age, body mass index (BMI), SES] and hospital of delivery.

For APIs in the State of Hawai‘i, Harvey, Lim, Gandhi, Miyamura, and Nakagawa (2017) found more cases of PPH among Asians (47.6%) and NHOPI (37.1%) compared to whites (15.3%). Other adverse obstetric outcomes such as prolonged labor, preeclampsia, placental abruption, and placenta previa were also associated with PPH. The study’s findings validate previous studies in that API women experience higher rates of PPH, independent of the known risk factors for PPH (e.g., maternal age, episiotomy rate, birth weight). Harvey et al.’s (2017) study is noteworthy when considering that the 2010 US census reported whites as the largest racial group in Hawai‘i (564,323), which is 40% greater than Filipinos (342,095), the second largest racial group (Fojas, Guevarra, & Sharma, 2018). Among non-English speakers in Hawai‘i (i.e., 93% of non-English languages spoken were from API regions), Sentell, Chang, Jun Ahn, and Miyamura (2016) found a two-fold risk of developing an obstetric trauma and pregnancy-related complications during vaginal birth, even after other factors were controlled.

Furthermore, three studies in the review investigated environmental exposures including volatile organic compounds (VOCs) and environmental tobacco smoke (ETS), and found disproportionate exposure levels for API women (Hoshiko et al., 2019; Williams et al., 2019). Williams et al.’s 2019 study found that nearly 51% of API women were exposed to significantly higher levels of VOCs in both the preconception and first-trimester windows compared to approximately 24% of white, black, and Hispanic women. Higher levels of VOC exposure were consistently observed to increase the odds of gestational diabetes mellitus (GDM) among API women, adding to the growing research on the associations between adverse pregnancy outcomes and preconception and prenatal VOC exposure. Similarly, Hoshiko et al. (2019) indicated that active smoking and ETS exposure patterns were discordant across groups. For example, Korean, Cambodian, and Vietnamese women had moderate to low active smoking, but high ETS exposure. These findings highlight environmental health disparities that are often overlooked due to small sample sizes and underscore the need for culturally and ethnically grounded interventions to advance public health practice.

Obstetric care and quality measures—such as vaginal birth after cesarean (VBAC) and episiotomy—were assessed by five studies. In particular, Janevic et al. (2018) found that only white women showed decreases in the incidence of obstetric quality measures [i.e., elective delivery (ED) before 39 weeks, low-risk cesarean delivery, and episiotomy], which raises concerns about why trends in the incidence of certain quality measures were observed among whites but not across other racial and ethnic groups. API women were more likely to have a VBAC than any other standard racial and ethnic group, however recent findings tend to obscure the heterogeneity within the API category (E. R. Cheng, Declercq, Belanoff, Iverson, & McCloskey, 2015; Edmonds, Hawkins, & Cohen, 2016). Five of the 22 reviewed articles evaluated risk factors for pregnancy-related complications (e.g., diabetes, prepregnancy obesity/overweight, chronic hypertension). Singh and DiBari (2019) revealed markedly high prepregnancy obesity levels among Hawaiians (32%) and Samoans (60%), with the highest prepregnancy overweight levels for Samoans (86.3%). Sociodemographic risk factors (e.g., maternal age, education, marital status, residence) only partially accounted for these racial and ethnic disparities in prepregnancy obesity. Miller et al. (2020) concluded that the risk of stroke varies for minority women, compared with white women, depending on a woman’s hypertensive status. Among the women with chronic hypertension, API women had a 17-fold higher risk of hemorrhagic stroke compared to white women. This was more than double the risk for black (6.57) and Hispanic women (6.90).

## Discussion

Racial and ethnic disparities in maternal health outcomes reflect differences in individual level sociodemographic factors (e.g., age, education, language, income, health behaviors, knowledge) to broad systemic factors (e.g., policy, access to high-quality care, racism and discrimination). While low SES underlies a significant portion of health disparities, this study’s literature review acknowledges that disparities in maternal outcomes, risk factors, and obstetric quality measures among API communities are not fully explained by SES variables. Several studies (Grobman et al., 2015; Singh & DiBari, 2019) found that even after controlling for socioeconomic factors, disparities in adverse maternal outcomes persisted. The review presents recent knowledge of the maternal health status for API women nationwide and suggests that API women are disproportionately affected by GDM, PPH, hypertension, and other maternal morbidities. With less than half of the reviewed studies disaggregating API data, there are important implications for health care systems as they shift toward patient-centered care. The need to comprehensively and accurately understand the health needs and health disparities of diverse APIs is ever-increasing. Racial and ethnic divides are expected to be further entrenched if research methodologies and health interventions neglect to meet API communities’ diverse health needs. Collective action is needed to reconceptualize marginalized API communities’ inclusion in health care systems, to promote their equitable access to care, and to dissolve health disparities among racial and ethnic divides.

The 2020 Social Progress Index—a comprehensive report developed by economists of the Social Progress Imperative—strengthens this call to action (*2020 Social Progress Index*, n.d.). By collecting data on 50 metrics (e.g., education, freedom, safety, health), the index compares the quality of life globally. Despite the country’s tremendous wealth, power, and cultural influence, the US now ranks 28th, a sharp decline from 19th during the last index in 2011. The US’ MMR—listed as a measure of basic medical care—is one of several areas in which the nation continues to underperform. The index depicts a complex and interconnected network consisting of fundamental human needs (e.g., shelter, safety, nutrition), foundations of well-being (e.g., access to knowledge, environmental quality), and opportunity (e.g., inclusiveness, personal rights) that must be considered when designing and implementing maternal health interventions for unique racial and ethnic populations.

There are several strengths that enhance the study’s credibility. First, the study provides a comprehensive analysis of four key areas in recent maternal health literature—and highlights a gap in research as regards identification of and engagement with the API category and/or its subgroups. Second, the study identifies findings that have been validated by previous studies and further supports a host of recommendations for reducing racial and ethnic disparities in maternal health outcomes. Third, the review examines studies that primarily draw upon data that were collected according to standardized procedures and thus the likelihood of observer bias is diminished. Also, there are several limitations to this study, which should be addressed in future research. First, less than half of the 22 reviewed studies disaggregated the large API category, meaning that unique health needs of different subgroups may be overshadowed. While the specified inclusion and exclusion criteria were able to substantially refine the search results, expanding the parameters of the criteria for future studies may allow greater access to disaggregated racial and ethnic data. Second, the API sample sizes in some of the studies were significantly smaller than other racial and ethnic groups, which may have resulted in nonstatistically significant results. Third, the inclusion and exclusion criteria may have stronger validity if an appropriate guiding instrument were to be applied. An extension of this study should incorporate a guiding instrument in order to enhance the inclusionary/exclusionary criteria of articles utilized in the data analysis process, such as the Melnyk Critical Appraisal Guide. Fourth, the literature review process did not include the identification of additional articles through other sources, as outlined in the PRISMA guidelines. Therefore, future research may consider incorporating a focused journal search in the literature identification phase.

## Recommendations

This paper provides the groundwork for future empirically-driven research across specific subgroups of the API category in the US broadly, and especially in Hawai‘i considering the rich multicultural nuances of Asian and Pacific Island communities. Studies that disaggregate APIs are limited, highlighting the need to understand the unique differences in maternal health and obstetric outcomes across all members of Hawai‘i’s diverse population. Stakeholders working to reduce racial and ethnic disparities in maternal health, especially among API women, should consider multifaceted interventions that target various contributing factors, ranging from broad upstream determinants to microlevel downstream determinants. Several pathways are recommended to enhance the development and implementation of effective and targeted health interventions for API groups. Such interventions can be addressed through short- and long-term initiatives.

Short-term initiatives may include developing community-based interventions that target maternal health education and use culturally relevant methods to effectively engage with the community. Similarly, building cultural competence for existing and emerging health care professionals is key to improving patient–provider relations. Cultural competence training and the implementation of readily available translator services in health care facilities would require consistent state leadership with private and public health partnerships. Long-term initiatives include addressing systemic sociocultural challenges between API communities and Western health institutions. A first step is to develop educational platforms that promote culturally grounded care. A second step to improve maternal health is through public policy, which is an extensive process and contingent on political priorities. Policy advocacy involves developing civic engagement and promoting stakeholders to testify in support of maternal health-related bills. A third step is to refine maternal health literature so that API communities are disaggregated from other racial and ethnic groups and understood on their unique conditions. A commitment to reducing disparities in maternal outcomes among API communities on both the local and global scale will yield momentous gains for public health and social justice.

## Acknowledgment

The authors acknowledge Dr. Emily Roberson’s contributions to this work.

## Declaration of Conflicting Interests

The authors declared no potential conflicts of interest concerning the research, authorship, or publication of this article.
